# Establishing a Dedicated Body Contouring Fellowship: Rationale, Structure, and a Framework for Subspecialty Training

**DOI:** 10.1093/asjof/ojag199

**Published:** 2026-10-28

**Authors:** Mohamed Badie Ahmed, Ahmed Al-Qahtani

## Abstract

Body contouring surgery has evolved into a complex area of plastic surgery that integrates aesthetic judgment, reconstructive planning, post-bariatric patient optimization, staged operative decision making, and long-term complication management. Despite increasing demand for post-massive weight loss and aesthetic body contouring procedures, dedicated fellowship pathways remain limited internationally, with most training embedded within broader aesthetic surgery fellowships. We describe the establishment of the Aesthetic and Reconstructive/Post-Massive Weight Loss Body Contouring Surgery Fellowship at Hamad Medical Corporation in Qatar, a structured 2 years program designed for graduates of plastic surgery residency programs seeking advanced clinical, operative, academic, and leadership experience in body contouring surgery. The fellowship includes progressive operative responsibility, multidisciplinary exposure, formal didactics, journal clubs, morbidity and mortality review, research, clinical audit, workplace-based assessment, and competency-based promotion. By comparing this model with existing international programs, particularly the University of Pittsburgh body contouring fellowship, we propose a practical framework for institutions seeking to develop dedicated body contouring training pathways. This model may be particularly relevant for regions where obesity, bariatric surgery, and post-weight-loss deformities are increasingly encountered but where formalized subspecialty training opportunities remain limited.

**Level of Evidence**: 5 (Therapeutic) 
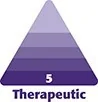

Body contouring surgery has become an increasingly important component of modern aesthetic and reconstructive plastic surgery. Excess skin after substantial weight loss is frequently observed, affecting up to 96% of patients following bariatric surgery. This condition can impair physical function and psychosocial well-being, limit daily activities, and often requires staged operative planning and individualized reconstruction.^1^ Although body contouring procedures are frequently included within plastic surgery residency and aesthetic surgery fellowship training, dedicated fellowships specifically focused on body contouring remain uncommon. In the United States, The Aesthetic Society–endorsed fellowships are generally 12-month programs focused on the breadth of aesthetic plastic surgery rather than body contouring alone.^2^ In contrast, the University of Pittsburgh offers a dedicated 12-month body contouring fellowship based in a high-volume multidisciplinary center focused on body contouring after obesity and weight loss, including body lifts, brachioplasty, thigh lifts, breast reshaping, and fat grafting.^3^

In the Middle East, the need for advanced body contouring training is increasingly relevant because of the growing burden of obesity, the expansion of bariatric surgery services, and rising demand for postbariatric and aesthetic body reshaping procedures. However, structured fellowship models dedicated to body contouring surgery remain scarce in the region. To address this gap, Dr Ahmad Al-Qahtani, a senior consultant in the Plastic Surgery Department at Hamad Medical Corporation (HMC), founding program director, and leading expert in the field, developed the Aesthetic and Reconstructive/Post-Massive Weight Loss Body Contouring Surgery Fellowship. This article describes the rationale, establishment process, structure, curriculum, assessment strategy, and distinguishing features of this fellowship. It also compares the HMC model with existing international fellowship pathways and proposes a framework that other institutions may adapt when developing similar subspecialty programs.

## RATIONALE FOR A DEDICATED BODY CONTOURING FELLOWSHIP

Body contouring surgery requires more than technical exposure to isolated procedures, such as abdominoplasty, liposuction, brachioplasty, or thigh lift. It requires advanced understanding of patient selection, body image concerns, massive weight loss physiology, nutritional optimization, staged operative sequencing, scar placement, gender-specific aesthetic proportions, complication prevention, and management of revision surgery. Therefore, a dedicated fellowship is needed for several reasons. First, post-massive-weight-loss patients frequently require complex, multiregional, and staged operations. Second, aesthetic outcomes depend heavily on individualized planning, soft-tissue assessment, scar design, and patient-specific goals. Third, body contouring patients may have psychological and body image concerns that require sensitive communication and multidisciplinary input.^4^ Fourth, safe practice requires familiarity with perioperative risks, wound complications, seroma, hematoma, infection, flap perfusion, implant-related issues, venous thromboembolism prevention, and revision strategies.^5-7^ Existing broad aesthetic fellowships provide valuable exposure to aesthetic plastic surgery; however, they may not always provide concentrated, longitudinal, and high-volume experience in postbariatric and complex body contouring. The Pittsburgh model demonstrates that body contouring can be organized as a focused fellowship pathway within a multidisciplinary post-weight-loss center. The HMC model builds on this concept while adapting it to the regional needs, institutional structure, and multidisciplinary resources available in Qatar.

A further institutional rationale for establishing this fellowship is the unique clinical volume generated by Qatar's public healthcare model.^8^ In Qatar, many cosmetic and body contouring procedures are provided free of charge for Qatari citizens in government hospitals, creating a high-volume environment for aesthetic and post-weight-loss body contouring surgery. It was highlighted that this model has resulted in substantial demand for cosmetic surgery services within the public sector, with ∼2000 aesthetic procedures performed annually across 4 governmental tertiary hospitals and nearly 90% of patients being Qatari nationals.^9^ This sustained patient flow provides an important educational foundation for fellowship-level training. In parallel, HMC has an established plastic surgery residency program with diverse case exposure, structured teaching, mentorship, and Accreditation Council for Graduate Medical Education-International (ACGME-I) accreditation, all of which have been identified by residents and program directors as important factors influencing training quality and specialty selection.^10^ Therefore, the proposed fellowship builds on 2 institutional strengths: a large body contouring patient population and a mature academic training environment. By creating a dedicated pathway in body contouring surgery, the program addresses an unmet service and educational gap while providing focused training for graduates whose interests extend beyond general plastic surgery residency toward advanced aesthetic and post-massive-weight-loss surgery.

## ESTABLISHMENT OF THE HMC BODY CONTOURING FELLOWSHIP

The HMC fellowship was developed as a structured 2 years program for graduates of recognized plastic surgery residency programs. The primary training institution is HMC, with Hamad General Hospital serving as the primary site along with multiple secondary sites. The establishment process followed several key steps: defining the educational gap, identifying institutional and regional need, developing a competency-based curriculum, selecting participating sites, defining core and advanced modules, incorporating academic and research requirements, and aligning assessment with ACGME-I-style competencies. The program accepts 1 to 2 fellows per year, allowing individualized supervision and progressive operative responsibility. A distinctive feature of the HMC fellowship is its integration of aesthetic and reconstructive body contouring rather than treating these as separate domains. This broad scope reflects the real-world overlap between aesthetic, reconstructive, functional, and psychosocial needs in post-massive-weight-loss patients.

## FELLOWSHIP STRUCTURE AND CURRICULUM

The fellowship is designed as a 2-year full-time supervised training program, with a possible third year for highly specialized technical development or external rotation (Figure 1). The first year focuses on advanced anatomy, patient selection, preoperative and postoperative care, principles of body contouring, liposuction, fat grafting, lifts, digital planning, 3-dimensional imaging, and abdominal wall and hernia surgery exposure. The second year focuses on advanced management of complex postbariatric cases, breast, gluteal, and facial contouring, minimally invasive techniques, and nonsurgical sculpting, with additional emphasis on leadership development, teaching skills, and completion of an individual research or scholarly project.

**Figure 1.**
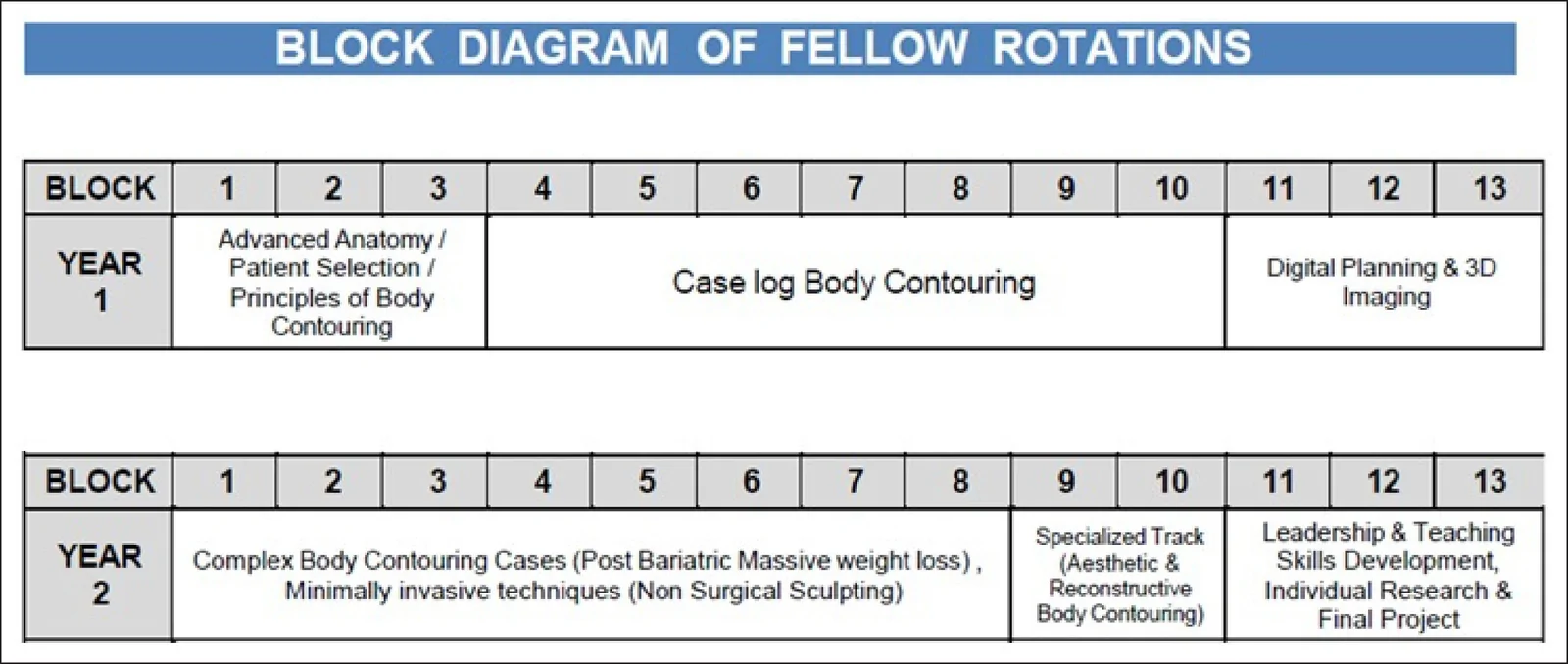
Two-year fellowship rotation block diagram showing Year 1 core modules and Year 2 advanced body contouring, specialized tracks, leadership, teaching, and research components.

The program follows the 6 general competencies used in graduate medical education: patient care, medical knowledge, practice-based learning and improvement, interpersonal and communication skills, professionalism, and system-based practice. These competencies are mapped to body contouring-specific goals and objectives, including staged operative planning, gender-specific aesthetic assessment, management of expectations, multidisciplinary communication, complication management, literature appraisal, quality improvement, and healthcare-system awareness. The fellowship also includes structured educational activities: morning report, didactic lectures, multidisciplinary courses and conferences, morbidity and mortality meetings, journal clubs, problem-based learning scenarios, grand rounds, research, and clinical audit (Figure 2). Fellows are provided protected time for these activities, and the educational program is integrated with the plastic surgery residency program.

**Figure 2.**
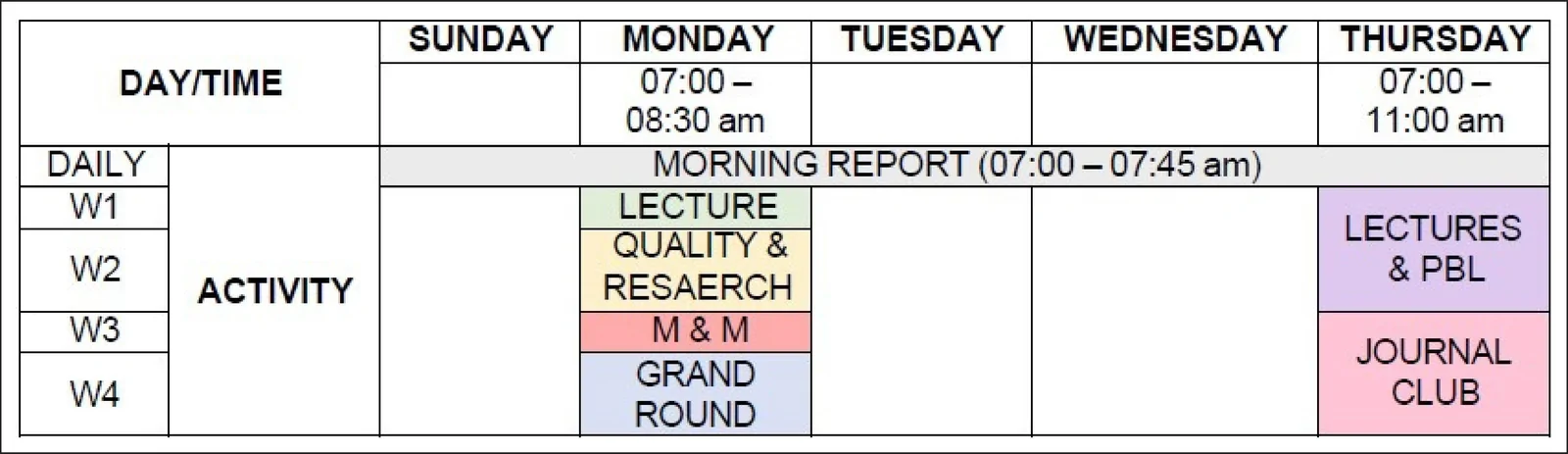
Weekly educational activity schedule showing morning reports, lectures, quality and research sessions, morbidity and mortality meetings, grand rounds, problem-based learning sessions, and journal club.

The program comprises 4 core faculty members, including the program director, and 4 off-site key faculty members. The core faculty includes 3 senior plastic surgery consultants, each with at least 10 years of experience in postmassive weight loss and body contouring surgery with diverse training backgrounds and international exposure from Sweden, Germany, and Qatar, and 1 senior specialist who has completed a fellowship in body contouring surgery in Colombia. The off-site key faculty comprises 3 senior plastic surgery consultants based at 3 different private institutions with high-volume plastic surgery practices, including post-massive-weight-loss body contouring and energy-based device procedures, and 1 general surgery consultant specializing in abdominal wall reconstruction and hernia repair. The senior plastic surgery consultants have extensive experience in the procedures specified in the fellowship logbook (Supplemental Figure 1). Together, these complementary areas of expertise support the program's training objectives. Faculty members are also internationally recognized through their scientific publications, invited lectures, and participation in major conferences. As the program was recently established, no fellows have graduated to date. Fellows cannot complete the program without meeting the minimum operative case requirements. A representative fellow logbook is provided in Supplemental Figure 1.

A major strength of the HMC model is its comprehensive competency-based assessment framework, which evaluates fellows through multiple tools, including formative evaluations, the Mini-Clinical Evaluation Exercise (Mini-CEX), Direct Observation of Procedural Skills (DOPS), teaching observation, logbook review, multisource feedback, patient surveys, summative assessments, and a final Objective Structured Clinical Examination–style clinical examination. Promotion is reviewed by a Clinical Competency Committee and is based not only on operative exposure but also on patient care, surgical skills, knowledge acquisition, professionalism, communication, research productivity, and all 6 general competencies. This approach is particularly important in body contouring surgery, where independent practice requires technical ability alongside sound judgment, operative planning, patient counseling, complication management, professional maturity, and academic contribution through mandatory research and publication.

## COMPARISON WITH EXISTING FELLOWSHIP MODELS

The HMC fellowship differs from many broad aesthetic surgery fellowships by offering a dedicated body contouring curriculum over 2 years rather than a 1-year general aesthetic experience. The Aesthetic Society–endorsed fellowships are 12-month programs focused on the breadth of aesthetic plastic surgery and follow an endorsed fellowship curriculum. Although these programs are valuable, their scope is intentionally broad and may include variable exposure to body contouring depending on institutional case mix and faculty expertise. The closest comparator is the University of Pittsburgh body contouring fellowship, which is described as a 1-year, clinical fellowship based in a high-volume multidisciplinary body contouring center and focused on body contouring after obesity and weight loss. Pittsburgh's fellowship provides advanced exposure to body lifts, brachioplasty, thigh lifts, breast reshaping, and fat grafting. The HMC model shares several similarities with Pittsburgh, including a dedicated focus on post-massive-weight-loss patients, multiregional body contouring procedures, fat grafting, breast reshaping, and multidisciplinary care.

However, the HMC model differs in several important ways. First, it is structured as a 2-year core fellowship with a potential third year for highly specialized training or external rotation, whereas Pittsburgh is described as a 12-month fellowship. Second, the HMC model explicitly integrates aesthetic and reconstructive body contouring, abdominal wall/hernia exposure, facial contouring, energy-assisted devices, research, clinical audit, and competency-based promotion. Third, the HMC fellowship is positioned within a public academic healthcare system in the Middle East, making it regionally adapted to local patient demographics, cultural expectations, healthcare pathways, and workforce needs.

## LESSONS LEARNED AND RECOMMENDATIONS FOR INSTITUTIONS

The HMC experience suggests several practical lessons for institutions aiming to develop body contouring fellowships. First, the program should be built around a clear needs assessment, including patient volume, case complexity, faculty expertise, and regional workforce gaps. Second, body contouring training should be multidisciplinary, incorporating bariatric surgery, nutrition, psychology or psychiatry, anesthesia, physical therapy, wound care, and abdominal wall reconstruction when relevant. Third, the curriculum should be competency-based and should include not only operative exposure but also patient selection, staged planning, complication management, communication, professionalism, research, and quality improvement. Fourth, programs should define progressive operative responsibility. The HMC model allows fellows to move from supervised participation to co-surgeon roles and eventual independent performance depending on competence. Fifth, programs should include objective assessment tools such as DOPS, Mini-CEX, logbooks, multisource feedback, patient surveys, and summative evaluations. Finally, research and audit should be embedded into the fellowship to promote academic development and generate local outcome data.

## CONCLUSIONS

The establishment of a dedicated body contouring fellowship at HMC addresses an important gap in subspecialty plastic surgery training not only in Qatar but in the Middle East as well. Although existing models such as the University of Pittsburgh fellowship demonstrate the feasibility of focused body contouring training in a high-volume post-weight-loss center, the HMC model offers a regionally adapted framework that integrates aesthetic and reconstructive goals within a public academic healthcare system. This fellowship may serve as a practical template for other institutions seeking to develop dedicated body contouring training programs.

## Supplementary Material

ojag199_Supplementary_Data
